# Yokoyama procedure for cyclic strabismus with axial high myopia:a case report

**DOI:** 10.1186/s12886-023-03257-w

**Published:** 2023-12-12

**Authors:** Min Ran, Kun-ming Cui, Yin Li, Xiu-sheng Song

**Affiliations:** Department of Ophthalmology, the Central Hospital of Enshi Tujia and Miao Autonomous Prefecture, Enshi Clinical College of Wuhan University, 445000 Enshi, Hubei Province China

**Keywords:** Cyclic strabismus, Axial high myopia, Yokayama procedure

## Abstract

**Background:**

It is a very rare form of ocular motility characterized by alternating strabismus and orthotropia. We report a patient with a 48-h cycle of esohypotropia associated with axial high myopia that resolved by Yokoyama procedure.

**Case presentation:**

A 43-year-old female patient who underwent left medial rectus muscle recession and lateral rectus muscle resection elsewhere due to highly myopic strabismus 2 years ago. The patient experienced a recurrence of left esohypotropia 12 months after undergoing surgery, exhibiting a 48-hour cycle. The cycle is one full day of esohypotropia and one day of orthotropia. The patient exhibited a case of high myopia in the left eye, characterized by a diopter measurement of -24.00DS and an eye axis measurement of 28.43 mm. Orbital CT showed supertemporal dislocation of the posterior portion of the elongated globe out from the muscle cone. Based on these observations, we performed Yokoyama procedure by uniting the muscle bellies of the superior rectus(SR) and lateral rectus (LR) muscles to restoring the dislocated globe back into the muscle cone.

**Conclusions:**

When cyclic strabismus is combined with axial high myopia, the Yokoyama procedure was effective and cycles are successfully terminated without overcorrection on no squint days. The purpose of this procedure is to put the dislocated globe back into its muscle cone by uniting the muscle bellies of the superior rectus and lateral rectus.

## Background

Cyclic strabismus is an infrequent ocular motility disorder distinguished by the alternating presence of strabismus and orthotropia. A majority of cyclic strabismus cases occur in children [1] and are idiopathic. It has been reported that adult-onset cyclic strabismus can be associated with central nervous system [2] and peripheral diseases, such as traumatic aphakia [3], optic atrophy [4], strabismus surgery [5, 6], high AC/A ratio [7] and dysthyroid ophthalmopathy [8], but no case of cyclic strabismus with axial high myopia has been reported. In spite of numerous attempts to explain the pathophysiology of this condition, the exact mechanisms remain unknown. In this study, we present a case of cyclic esohypotropia with axial high myopia who underwent medial rectus (MR) recession and lateral rectus (LR) muscle resection. As the patient’s orbital CT showed supertemporal dislocation of the posterior portion of the elongated globe out from the muscle cone, we performed Yokayama procedure on her left eye with good results.

## Case presentation

**Fig. 1:**
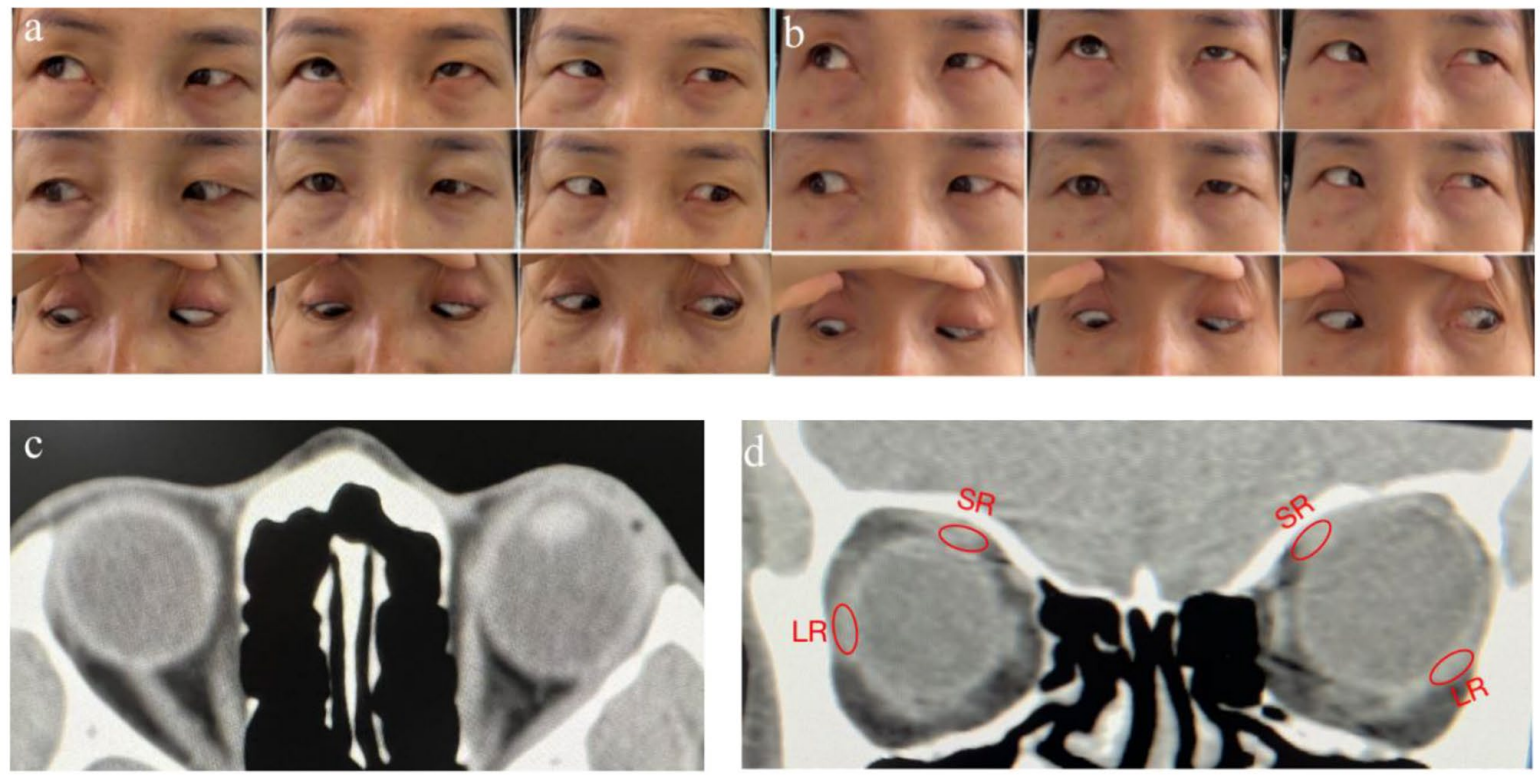
Patient’s photographs on a “bad” day and a “good” day, plus computed tomography (CT)scans. **a** “Bad” day.The patient manifested a 60 PD esotropia and 30 PD hypotropia in primary position with mild limitation of abduction in her left eye. **b** Orthotropia on “Good” day. **c** and **d** supertemporal dislocation of the posterior portion of the elongated globe out from the muscle cone in her left eye on CT scan

A 43-year-old woman presented esohypotropia with axial high mypia in her left eye four years ago ,she underwent medial rectus(MR)muscle recession and lateral rectus (LR)muscle resection on her left eye 2 years ago elsewhere. Twelve months after surgery, she developed recurrence of left eye esohypotropia with a cycle of squinting one day (Fig. 1a) and not squinting the next (Fig. 1b). Her right best-corrected visual acuity was 20/20 with a refractive error of -0.75 diopter sphere/-2.25 diopter cylinder at 117°, Her left best-corrected visual acuity was CF/40 cm with a refractive error of -24.00 diopter sphere/-2.00 diopter cylinder at 115°. Due to myopic maculopathy, her left eye has poor corrected vision. The worth 4-dot test demonstrated left suppression on both squint and non-squint days. The axial lengths of her right and left globe were 22.35 and 28.37 mm respectively. She exhibited left esotropia of 60 prism diopters (PD) and hypotropia of 30 PD in the primary position with mild limitation of left eye abduction (Fig. 1a).Orbital CT showed supertemporal dislocation of the posterior portion of the elongated globe out from the muscle cone(Fig,1c and d).

**Fig. 2:**
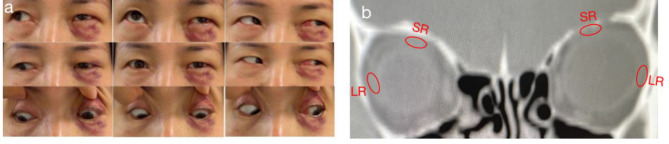
Clinical photographs and CT scan of the patient showing ocular alignment after surgery. **a** Orthotropia on the postoperative day 7 **b** Orbital CT showed dislocated globe back into the muscle cone

We examined the patient six times over 6 months. Eight months after the onset of the cyclic phase, the patient underwent extraocular muscle surgery based on the full measurement on a squint day. During surgery, the forced duction test (FDT) revealed no restriction in her left eye. We found the distance from the limbus to the insertion of the medial rectus muscle was 10 mm. We performed left medial rectus recessions of 12 mm from the limbus combined with uniting the muscle bellies of the superior rectus(SR) and lateral rectus (LR) muscles to restoring the dislocated globe back into the muscle cone. On the first week postoperative day (Fig. 2a), the patient was orthotropia. Orbital CT showed dislocated globe back into the muscle cone(Fig. 2b). At 6 months of postoperative follow-up, no recurrence of esohypotropia was observed.

## Discussion

A cyclic strabismus is characterized by periodic alternating strabismus and orthotropia. The most common manifestation is a 48-h cycle consisting of a 24-h period of strabismus followed by a 24-h period of orthotropia. Cycles with 72-h, 96-h and monstrual periods may also occur [9, 10]. Cyclic strabismus is not known to have a mechanism, although biological clocks and diurnal and circadian rhythms may play a role [1, 2, 11]. For cyclic pattern, following theories have been proposed in the literature – aberration of the central nervous system disease [2], peripheral disease such as traumatic aphakia[3], optic atrophy [4], strabismus surgery [5, 6], high AC/A ratio [7] and dysthyroid ophthalmopathy [8]. Children with cyclic strabismus usually begin experiencing it around 3 to 4 years of age without any triggering events. In the strabismus phase, patients have poor stereopsis and suppression. Cyclic strabismus can occur after visual maturity due to conditions disrupting fusion or causing diplopia [4]. In our present case, due to the enlarged left globe herniation through the supertemporal muscle cone associated with axial high myopia, binocular fusion was disrupted, which may have triggered the onset of the cyclic esohypotropia.

There have been reports of high myopic patients developing horizontal and vertical strabismus during adulthood, generally after the third decade. Symptoms range from small-angle esotropia with mild abduction deficit to severe restriction [12, 13]. A variety of surgical procedures are used in traditional surgery, such as tenotomy or recession of the medial rectus muscle, tenotomy of the nasal conjunctiva, regular resections and recessions, and traction sutures [13, 14]. In our present patients, her left eye was esohypotropic with mild abduction deficit, ordinary recession and resection procedure that performed 2 years ago was effective, but it relapsed 12 months later. The axial length (AL) of left globe is 28.43 mm. CT scan showing supertemporal dislocation of the posterior portion of the elongated globe out from the muscle cone. Probable reason for relapse of highly myopic strabismus in our case was the result of redislocation of the globe after temporary placement back within the muscle cone by medial rectus muscle recession and lateral rectus muscle resection.

The most common treatment about cyclic strabismus is extraocular muscle surgery based on the maximal deviation measured on “squint” days. Also reported are botulinum injections [15] and prismatic corrections [16]. In our case, due to supertemporal dislocation of the posteriormost portion of the globe out of the muscle cone, we performed Yokoyama procedure by uniting the superior and lateral rectus muscles without scleral suture plus medial rectus recession. This procedure was effective and successfully terminates the cycle without overcorrection on no squint days. At 6 months of postoperative follow-up, no recurrence of esohypotropia was observed.

In summary, cyclic strabismus is a rare condition of unknown origin. We first report Yokoyama procedure for cyclic strabismus with axial high myopia, the Yokoyama procedure was effective and successfully terminates the cycle without overcorrection on no squint days.The purpose of this procedure is to put the dislocated globe back into its muscle cone by uniting the muscle bellies of the superior rectus and lateral rectus.

## Data Availability

All data generated or analyzed during this study are included in this published article.
